# Treatment of Hyperoxia-Induced Lung Injury with Lung Mesenchymal Stem Cells in Mice

**DOI:** 10.1155/2018/5976519

**Published:** 2018-09-26

**Authors:** Yabo Mei, Chong Chen, Hui Dong, Wanqiao Zhang, Yan Wang, Ming Chi, Zhichun Feng

**Affiliations:** ^1^Department of Pediatrics, Affiliated BaYi Children's Hospital, PLA Army General Hospital, Beijing, China; ^2^The National Engineering Laboratory for Birth Defects Prevention and Control of Key Technology, Beijing, China; ^3^Beijing Key Laboratory of Pediatric Organ Failure, Beijing, China; ^4^Department of Pediatrics, The First Affiliated Hospital of Chinese PLA General Hospital, Beijing, China; ^5^Neonatal Intensive Care Unit of Jining No. 1 People's Hospital, Jining, Shangdong Province, China

## Abstract

**Objective:**

Bronchopulmonary dysplasia (BPD) is a common chronic lung disease in preterm neonates and has no effective treatment. This study aimed to investigate the therapeutic effects of neonatal mouse lung resident mesenchymal stem cells (L-MSCs) on the hyperoxia-induced lung injury.

**Methods:**

L-MSCs were separated and identified according to the MSC criterions. Hyperoxia-Induced Lung Injury (HILI) of neonatal KM mice was induced with hyperoxia (FiO_2_ = 60%) and investigated with pathological methods. Neonatal KM mice were divided into 3 groups (hyperoxia + L-MSC group, hyperoxia + PBS group, and air control group). Mice in the hyperoxia + L-MSC group were treated with L-MSCs at 3, 7, and 14 days after birth. After hyperoxia exposure for 21 days, the lung pathology, Radial Alveolar Count (RAC), CD31 expression, and vascular endothelial growth factor (VEGF) expression were investigated.

**Results:**

After hyperoxia exposure, the body weight, RAC, CD31 expression, and VEGF expression in the hyperoxia + L-MSC group were significantly better than those in the hyperoxia + PBS group but inferior to those in the air control group significantly. These indicate L-MSCs are partially protective on the lung injury of mice with hyperoxia-induced BPD.

**Conclusion:**

L-MSCs are helpful for the prevention and treatment of BPD, and endogenous L-MSCs may play a role in the postinjury repair of the lung.

## 1. Introduction

Bronchopulmonary dysplasia (BPD) is a specific chronic respiratory disease in neonates and refers to the developmental arrest of the immature lungs due to some pathological factors. The pathology of BPD is characterized by the reduced amounts of alveoli and capillaries, enlargement and structural simplification of alveoli, and basement membrane thickening, and alveolar and pulmonary microvascular dysplasia are a major pathological feature of BPD [1]. BPD has been extensively studied since it was reported for the first time in 1967 by Northway et al. [2]. However, the pathogenesis of BPD is still poorly understood so far, and effective strategies have not been developed yet for the prevention and treatment of BPD. Cellular therapies using exogenously mesenchymal stem cells (MSCs), as alternative therapies, may represent the next breakthrough therapies for the treatment of BPD and have attracted growing attention in the past decade [3].

The improvement in lung function and architecture had been verified in a growing body of animal models received exogenously MSCs or MSC-conditioned media [4–6], but very little is known about that of lungs' own endogenous MSCs [4, 5, 7]. L-MSCs can be isolated from the neonatal lung and adult lung of the mouse or human just like bone-marrow MSCs and other tissue-derived MSCs [7]. As lung-resident MSCs, L-MSCs producing specific basement membrane and promoting epithelial progenitor cells' proliferation show organ-specific characteristics [8] and are functionally distinct from BM-MSCs and specifically geared for the pulmonary environment [7, 9]. L-MSCs exhibit the same protective effect as BM-MSCs for elastase-induced lung injury but greater survival and higher lung retention after intravenous injection in a mouse model [9]. In addition, exogenous administration of additional L-MSCs attenuating the experimental lung fibrosis in a mouse model suggested that replenishing L-MSCs may be protective against lung injury factors [10].

However, on the other hand, L-MSCs may exert fibrotic effects to induce lung fibrosis and be involved in the pathogenesis of some lung diseases under certain conditions [2, 7, 11]. L-MSCs separated from the tracheal aspirate of preterm neonates [12] and increased TGF-*β* in the blood and tracheal aspirate [13, 14] are closely related to the development of BPD. There is evidence showing that L-MSCs are important for alveolarization by strictly regulating alveoli septum and blood vessel formation [7]. The destruction of this process may cause the simplification of alveolar structure and abnormality in the lung vasculature, resulting in pulmonary hypertension, which is a key feature of “new BPD” [2]. BPD is characterized by a widespread increase of myofibroblasts [15, 16], and moreover, the behavior of L-MSCs in BPD is altered and leans toward a myofibroblast phenotype [7]. These indicate that L-MSCs are associated with the occurrence and development of BPD.

These studies show the heterogeneity of L-MSC functionality under different conditions. A possible explanation is that the L-MSCs isolated from BPD had been induced and lean toward a myofibroblast phenotype but the primitive L-MSCs have not been induced and show the protective effect against hyperoxia injury. To date, few studies have been conducted to investigate the functions of L-MSCs [2, 7]. Thus, investigating the protective effects of L-MSCs on hyperoxia-induced lung injury (BPD) may provide new treatment for BPD and is also helpful for the elucidation of the pathogenesis of BPD. In this study, the in vitro L-MSCs from neonatal KM mice were used to treat neonatal KM mice with hyperoxia-induced BPD. During hyperoxia, mice were intraperitoneally injected with additional neonatal mouse L-MSCs; the alveolar and microvascular formations were compared between the L-MSC group and control group, aiming to evaluate the protective effects of L-MSCs on the alveolar and microvascular formations. This study may elucidate the protective effects of L-MSCs on the BPD and provide new strategies for the prevention and treatment of BPD in preterm neonates.

## 2. Methods

### 2.1. In Vitro Culture and Identification of Neonatal Mouse L-MSCs

The separation, culture, and identification of neonatal mouse L-MSCs were done according to the method reported elsewhere [17], which was recorded in detail in the supplementary materials (available here).

### 2.2. Establishment of Hyperoxia-Induced BPD in Neonatal Mice

Establishment of hyperoxia-induced BPD in neonates was done as recorded in the supplementary materials as described previously [18].

### 2.3. Treatment of Hyperoxia-Induced Lung Injury with Mouse L-MSCs

#### 2.3.1. Animal Feeding

A total of 30 Kunming mice (specific pathogen free) within 24 h after birth were randomly assigned into 3 groups: air control group, hyperoxia + L-MSC group, and hyperoxia + PBS group (*n* = 10 per group). In the hyperoxia + L-MSC group and hyperoxia + PBS group, mice were placed in a closed box which was flushed with oxygen at a high concentration (FiO_2_ 60%). In the air control group, mice were exposed to air. Animals received normal food, and the bedding and sodium lime were refreshed regularly to assure the clean environment. Mice were housed with their mother. The mother mice in the BPD groups were exchanged with those in the control group once every 24 h to avoid the poor feeding due to oxygen poisoning and the consequent slow growth of neonate mice. At 3, 10, and 17 days after birth, mice in the hyperoxia + L-MSC group received intraperitoneal injection with L-MSCs (10^5^); mice in the hyperoxia + PBS group received intraperitoneal injection with PBS of equal volume. At 21 days after birth, mice were intraperitoneally anesthetized with chloral hydrate, and lung tissues were collected after thoracotomy, washed in PBS, and then placed in 4% paraformaldehyde or stored in liquid nitrogen.

#### 2.3.2. Sample Processing

*(1) HE Staining*. Lung tissues were fixed in 4% paraformaldehyde for 12–24 h, dehydrated in the ethanol at a series of concentrations, transparentized in xylene, embedded in paraffin, and sectioned (5 *μ*m). Sections were collected onto slides which were then heated at 35–40 °C. After deparrafinization, sections were stained with hematoxylin and eosin. After dehydration in ethanol and transparentization in xylene, mounting was done, and sections were observed under a light microscope.

*(2) Radial Alveolar Count (RAC)*. After HE staining, sections were observed under a light microscope at a magnification of 100×. A line was made between the center of the bronchiole and the nearest fiber septum or pleura, and the alveoli were counted along the line, which reflects the degree of alveolarization and the extent of lung development/injury. At least 3 sections were evaluated for each mouse, counting was done 5 times for each section, and a mean was obtained as the RAC.

*(3) Immunohistochemistry*. Sections were treated with xylene and then dehydrated in ethanol at a series of ethanol, followed by antigen retrieval. After washing in PBS, sections were incubated with 3% hydrogen peroxide for 15 min at room temperature to inactivate endogenous peroxidase. After washing in PBS, sections were treated with diluted primary antibody at 4 °C in a humidified box. On the second day, reagent 1 and reagent 2 (immunohistochemistry test kit; Zhongshan Jinqiao Biotechnology Co. Ltd., China) were added to each section, followed by addition of freshly prepared DAB solution. Sections were then observed under a light microscope, and positive cells were brown-yellow. Counterstaining was done with hematoxylin. After washing, sections were treated with 1% hydrochloric acid in ethanol. After washing, sections were dehydrated in ethanol at a series of concentrations and then air-dried. Mounting was done with neutral gum, and sections were subsequently observed and photographed under a light microscope. Image-Pro Plus 6.0 was used to determine the mean optical density of CD31 expression in the lung.

*(4) Statistical Analysis*. Statistical analysis was performed with SPSS version 19.0. Data are expressed as mean ± standard deviation. Comparisons were done with independent sample *t*-test between groups or one-way analysis of variance among groups. A value of *P* < 0.05 was considered statistically significant.

## 3. Results

### 3.1. L-MSC Injection Partially Offsets the Effect of Hyperoxia on Body Weight Gain in Mice

**Figure 1:**
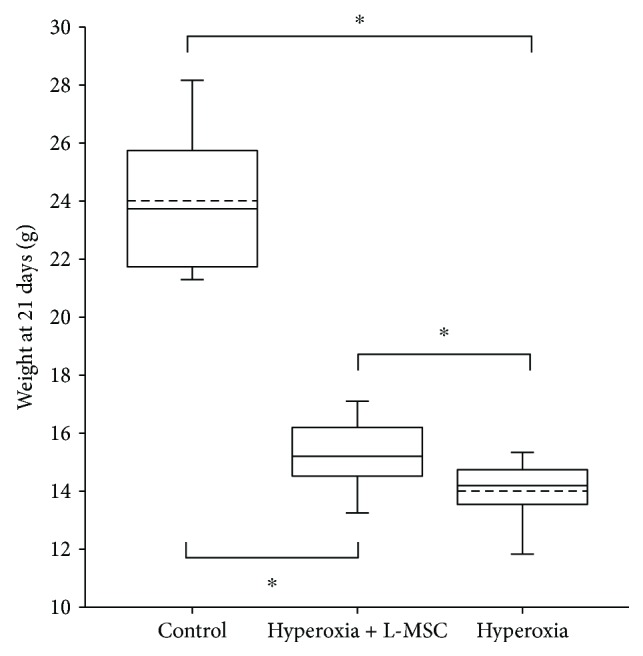
Body weight of mice in different groups. The body weight of the air control group, hyperoxia + PBS group, and hyperoxia + L-MSC group was 24.01 ± 2.46 g, 14.01 ± 1.04 g, and 15.91 ± 0.93 g. Significant difference in the body weight was observed among the three groups (*F* = 105.87, *P* = 0.000). The body weight of the hyperoxia + L-MSC group was higher that of the hyperoxia + PBS group (*P* = 0.038) and lower than that of the air control group (*P* = 0.000) significantly. *n* = 10 per group; dotted line: mean; ^∗^ *P* < 0.05.

Hyperoxic exposure can have a significant negative effect on body weight gain in mice. As showed in Figure 1, results showed that the body weight was the highest in the air control group and the lowest in the hyperoxia + PBS group, and significant difference in the body weight was observed among the three groups (*P* < 0.001). The body weight of the hyperoxia + L-MSC group was higher than that of the hyperoxia + PBS group (*P* < 0.05) and lower than that of the air control group (*P* < 0.001) significantly.

### 3.2. L-MSCs Increase RAC and Enhance VEGF/CD31 Expression of the Lung under Hyperoxic Exposure

#### 3.2.1. Additional L-MSC Supplementation Improves the Morphological Changes of the Hyperoxia-Induced Lung

**Figure 2:**
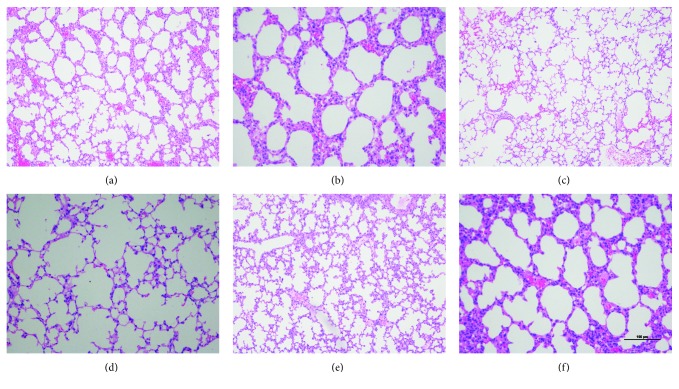
HE staining of lung tissues: (a) air control group (100×); (b) air control group (200×); (c) hyperoxia + PBS group (100×); (d) hyperoxia + PBS group (200×); (e) hyperoxia + MSC group (100×); (f) hyperoxia + MSC group (200×). Scale bar = 100 *μ*m and the 6 pictures have the same magnification.

To analyze the repair effect of L-MSCs on hyperoxia-induced lung injury, we analyzed the pathological sections of lung tissue from three groups of experimental animals. After HE staining of lung tissues, sections were observed under a light microscope. In the air control group (Figures 2(a) and 2(b)), the lung structure was complete, a lot of alveoli were observed, and alveoli had regular shape and even size. In the hyperoxia + PBS group (Figures 2(c) and 2(d)), the normal lung structure was missing, the alveolar space was enlarged, the amount of alveoli was reduced, alveoli merged to form large alveolar septa, and some alveolar septa were disrupted, which were the characteristics of BPD. In the hyperoxia + L-MSC group (Figures 2(e) and 2(e)), the lung structure was more similar to that in the air control group (Figure 2).

#### 3.2.2. Additional L-MSC Supplementation Increases the RAC of the Hyperoxia-Induced Lung

**Figure 3:**
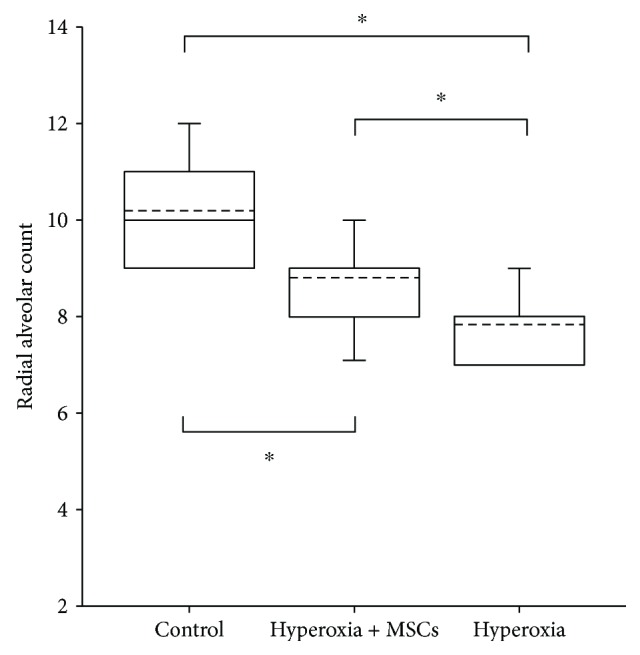
RAC of mice in different groups. The RAC of the air control group, hyperoxia + PBS group, and hyperoxia + L-MSC group were 10.2 ± 1.14, 7.83 ± 0.98, and 8.81 ± 0.96, respectively. Significant difference in the body weight was observed among the three groups (*F* = 217.40, *P* = 0.000). The RAC of the hyperoxia + L-MSC group was higher than that of the hyperoxia + PBS group (*P* = 0.000) and lower than that of the air control group (*P* = 0.000) significantly. *n* = 10 per group; dotted line: mean; ^∗^ *P* < 0.05.

Hyperoxia exposure arrests the development of the immature lung and reduces lung RAC. Our results showed RAC was the highest in the air control group and the lowest in the hyperoxia + PBS group, and significant difference in RAC was observed among the three groups (*P* < 0.001). The RAC of the hyperoxia + L-MSC group was higher than that of the hyperoxia + PBS group (*P* < 0.001) and lower than that of the air control group (*P* < 0.001) significantly (Figure 3).

#### 3.2.3. Additional L-MSC Supplementation Enhances the VEGF Expression of the Hyperoxia-Induced Lung

**Figure 4:**
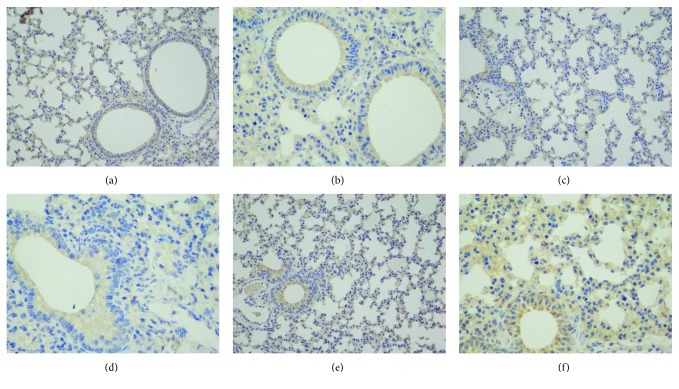
VEGF expression in the lung: (a) air control group (200×); (b) air control group (400×); (c) hyperoxia + PBS group (200×); (d) hyperoxia + PBS group (400×); (e) hyperoxia + MSC group (200×); (f) hyperoxia + MSC group (400×).

**Figure 5:**
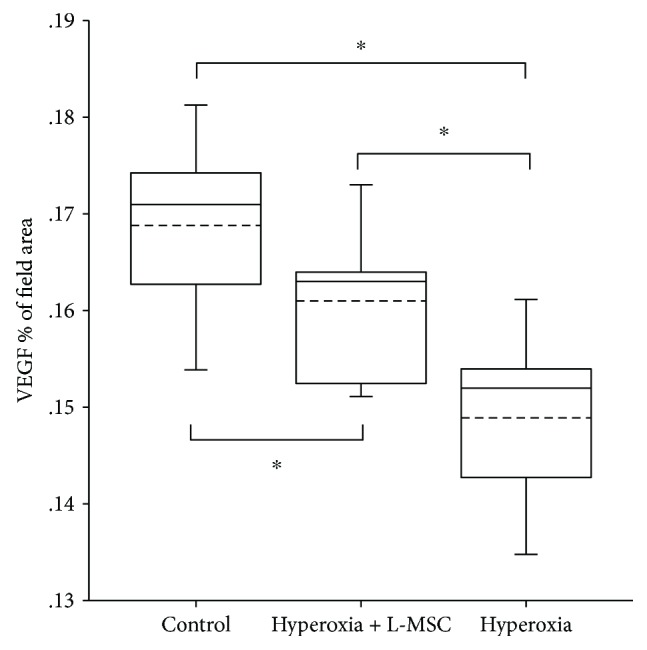
VEGF expression in the lung of mice in different groups. The optical density of VEGF expression was 0.169 ± 0.008 in the air control group, 0.161 ± 0.007 in the hyperoxia + L-MSC group, and 0.149 ± 0.008 in the hyperoxia + PBS group. There was marked difference in the VEGF expression among the three groups (*t* = 16.270, *P* = 0.000). The VEGF expression of L-MSC treatment (E and F) was higher than that of the hyperoxia + PBS group (*P* = 0.035) but lower than that of the air control group (*P* = 0.035) significantly. *n* = 10 per group; dotted line: mean; ^∗^ *P* < 0.05.

The VEGF expression in the lung of mice was detected by immunohistochemistry. Results showed VEGF was mainly expressed in the alveolar septa and airway epithelium. Compared to the air control group (Figures 4(a) and 4(b)), VEGF expression was inhibited by hyperoxia exposure (Figures 4(c) and 4(d)). However, additional L-MSC supplementation enhances the VEGF expression of the hyperoxia-induced lung. The optical density of VEGF expression analysis confirmed the protection of L-MSCs. There was marked difference in the VEGF expression among the three groups (*P* < 0.001). The VEGF expression of L-MSC treatment (Figures 4(e) and 4(f) was higher than that of the hyperoxia + PBS group (*P* < 0.05) but lower than that of the air control group (*P* < 0.05). (Figures 4 and 5).

#### 3.2.4. Additional L-MSC Supplementation Enhances the CD31 Expression of the Hyperoxia-Induced Lung

The CD31 expression in the lung of mice was detected by immunohistochemistry.

Results showed CD31 was mainly expressed in the endothelial cells of major vessels and microvessels.

**Figure 6:**
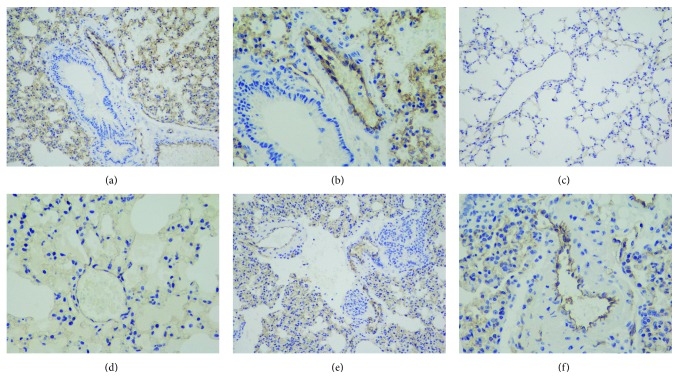
CD31 expression in the lung: (a) air control group (200×); (b) air control group (400×); (c) hyperoxia + PBS group (200×); (d) hyperoxia + PBS group (400×); (e) hyperoxia + MSC group (200×); (f) hyperoxia + MSC group (400×).

**Figure 7:**
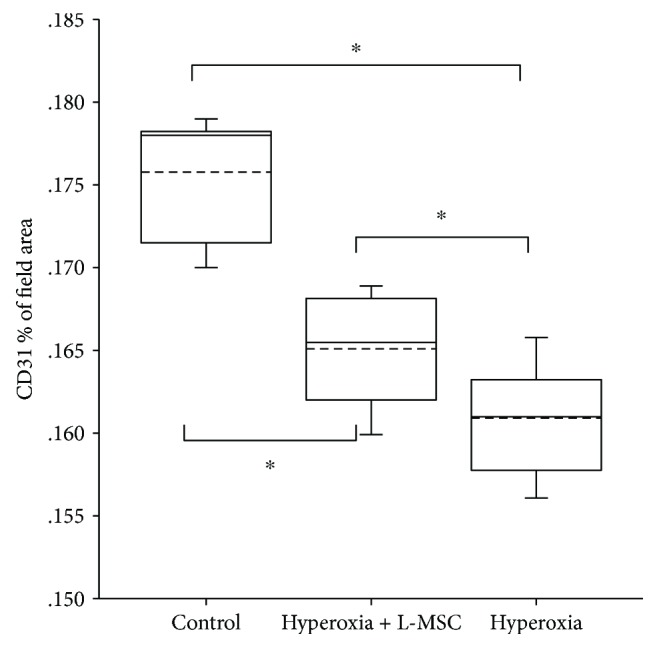
CD31 expression in the lung of mice in different groups. The optical density of CD31 expression was 0.176 ± 0.004 in the air control group, 0.165 ± 0.003 in the hyperoxia + L-MSC group, and 0.161 ± 0.003 in the hyperoxia + PBS group. There was marked difference in the CD31 expression among the three groups (*F* = 57.866, *P* = 0.000). The CD31 expression of L-MSC treatment (E and F) was higher than that of the hyperoxia + PBS group (*P* = 0.014) but lower than that of the air control group (*P* = 0.014) significantly. *n* = 10 per group; dotted line: mean; ^∗^ *P* < 0.05.

Compared to the air control group (Figures 6(a) and 6(b)), CD31 expression was inhibited by hyperoxia exposure (Figures 6(c) and 6(d)). However, additional L-MSC supplementation enhances the CD31 expression of the hyperoxia-induced lung. The optical density of CD31 expression analysis also confirmed the proangiogenic effect of L-MSCs under hyperoxia exposure. There was marked difference in the CD31 expression among the three groups (*P* < 0.001). The CD31 expression of L-MSC treatment (Figures 6(e) and 6(f)) was higher than that of the hyperoxia + PBS group (*P* < 0.05) but lower than that of the air control group (*P* < 0.05). (Figures 6 and 7).

## 4. Discussion

BPD is a common complication in preterm neonates, especially the very low birth weight infants and clinically characterized by oxygen dependence. BPD is closely related to several adverse outcomes such as death, cerebral palsy, cognitive dysfunction, and growth and development disorders and has been found as an independent risk factor of neurodevelopmental disorders [19]. BPD is caused by different factors and is mainly regarded as the acute injury to the premature lung due to several pathogenic factors. These factors (such as inflammation, hyperoxia, barotraumas, and volutrauma) act synergistically to cause damage to the immature lung. Although the BPD has been studied for more than 50 years, the pathogenesis of BPD is still poorly understood. Thus, it is difficult to develop effective strategies for the prevention and treatment of BPD.

The typical BPD is rare with the prenatal use of hormone and postnatal use of pulmonary surfactant. The “new” BPD is pathologically characterized by the alveolar and lung microvascular dysplasia. RAC may directly reflect the difference in the alveolar formation. CD31 is a surface marker of endothelial cells and a marker of the vascular bed. CD31 expression may reflect the lung microvascular development. In lung development, VEGF is important for lung vascularization, parenchyma maturation, and surfactant production [20, 21]. VEGF is mainly derived from ACE-II and is a potent mitogen acting on vascular endothelial cells [22]. VEGF plays an important role in the angiogenesis and vascularization in the embryonic phase [23]. In addition, VEGF can inhibit apoptosis to improve endothelial survival. The reduction in VEGF expression reflects the reduced perfusion, which may compromise the gas exchange in the lung [22]. Thus, the RAC, CD31 expression, and VEGF expression may reflect alveolar and microvascular development and are also commonly used in the investigations on BPD [6].

A lot of preclinical studies confirm the effectiveness and safety of MSCs in the treatment of BPD [6], but little is known about the clinical treatment of BPD with MSCs [24]. L-MSCs are not only the localized mesenchymal progenitor cells but also the lung-specific regulatory cells in the alveolar development/lung microvascular formation [7–9, 25]. As lung resident MSCs, L-MSCs seemingly have more advantages for lung injury compared to MSCs derived from other tissues [7–10]. Studies have shown that L-MSCs can promote the growth of alveolar type II epithelial cells (AEC-II) [26]. Lipofibroblasts are derived from L-MSCs and can secrete prostaglandin-E2 and thyroxine-related protein to transport triglyceride into AEC-II, leading to the early synthesis of pulmonary surfactants [27]. Lipofibroblasts can also store and secrete retinoic acid, which may also promote the alveolar septal and alveolar development [26]. In addition, in vitro studies indicate that coculture of AEC-II and lipofibroblasts may promote the formation of small alveolar structures [28]. These studies suggest that L-MSCs can promote the development and regeneration of lung tissues in neonates [29].

In the present study, neonatal mice were exposed to hyperoxia to induce BPD, which has been widely used to establish the BPD animal model [21]. In our study, animals were exposed to hyperoxia for 21 days to induce BPD and animals exposed to air served as negative controls. L-MSCs were intraperitoneally injected for the treatment. The body weight, RAC, CD31 expression, and VEGF expression were determined. Results showed hyperoxia exposure could significantly reduce the body weight and lung RAC as well as inhibit CD31 expression and VEGF expression. However, intraperitoneal injection of L-MSCs could markedly improve body weight, RAC, CD31 expression, and VEGF expression in mice exposed to hyperoxia. This indicates intraperitoneal L-MSCs may antagonize the adverse consequence of long-term hyperoxia to the immature lung. As compared to hyperoxia-exposed mice, the lung RAC increased significantly, and the alveolar structural integrity, septal development, VEGF expression, and CD31expression were also markedly improved. This suggests that intraperitoneal injection of L-MSCs is able to promote the alveolar and lung vascular development of mice exposed to hyperoxia. The VEGF is mainly produced in AEC-II in the lung [21], and our results show that the VEGF expression in the mouse lung receiving L-MSC injection was significantly higher than that in the positive control group, indicating that intraperitoneal injection of L-MSCs can improve the functions of intrapulmonary AEC-II in the injured lung.

Sajit Augustine et al. conducted a meta-analysis about the treatment of BPD with MSCs in animal studies. Their results showed that the efficacy of MSCs had little relationship with the route and dose of MSCs injected. Another study of van Haaften et al. [29] showed that treatment at 4 days after birth could achieve better efficacy as compared to treatment at 14 days after birth in animals with hyperoxia-induced lung injury. This indicates that hyperoxia exposure for 3 days may induce damage to immature lung tissues, causing long-term BPD. Thus, in the present study, treatment was administered twice at 3 days after birth, which may achieve a better efficacy. As a consequence, our results showed that L-MSCs can effectively alleviate the lung injury in BPD mice and promote microvascular regeneration and alveolar development, which then improve the general condition and survival. Our results also indicate that L-MSCs play an important role in the repair of hyperoxia-induced lung injury and may be used as an alternative source of cell therapy for lung injury. Moreover, on the basis that human L-MSCs have been successfully isolated from bronchoalveolar lavage fluid of neonates as described previously [12], the isolation of primitive L-MSCs may be possible from the neonate who received ventilation therapy soon after birth, which would provide a new direction for the clinical treatment of BPD.

On the basis of our results, L-MSCs only partially improve the hyperoxia-induced lung injury, which suggest the incomplete efficacy of L-MSCs. Whether multiple injections, a higher dose of L-MSCs, and other routes of L-MSC injection may further improve the efficacy is warranted for investigation in more studies. In addition, previous studies have shown that the gene expression profile of L-MSCs is different from that of MSCs from other tissues or organs [8]. Considering the important role of L-MSCs in the lung development, future studies are needed to investigate the specific mechanisms underlying the therapeutic effects of L-MSCs and whether these mechanisms are also found in the effects of MSCs derived from other tissues or organs.

## Data Availability

The data used to support the findings of this study are available from the corresponding author upon request.

## Supplementary Materials

Supplementary Materials The separation, culture, and identification of neonatal mouse L-MSCs and establishment of hyperoxia-induced BPD in neonatal mice were described in details in supplementary materials.
