# Supplementary material for: Treatment of Hyperoxia-Induced Lung Injury with Lung Mesenchymal Stem Cells in Mice
Source: Stem Cells Int. 2018 Sep 26;2018:5976519. doi: 10.1155/2018/5976519 (PMC6178508; doi:10.1155/2018/5976519)
Supplement: Supplementary Materials — The separation, culture, and identification of neonatal mouse L-MSCs and establishment of hyperoxia-induced BPD in neonatal mice were described in details in supplementary materials. [file 5976519.f1.pdf]

Part 1. Establishment of BPD model

Methods

We purchased newborn Kunming mice from the experimental animal center of the Academy of Military Medical Sciences of the Chinese PLA (Beijing, China) and randomly assigned 20 to room air and 20 to hyperoxia (FIO2 60% for 21 days), beginning at birth. Nursing dams were alternated every 24 hours between the air and hyperoxia litters. All the mice were maintained in a pathogen-free facility and used in accordance with our institutional guidelines for animal care. 5 animals randomly selected were euthanized with intraperitoneal sodium pentobarbital after exposure on day 3, day 7, day 14 or day 21. The body weight and RAC of animals were analyzed.

Rsults

Effects of 60% Oxygen Exposure on Newborn Mice

There was no mortality in either group, but the hyperoxia group had significantly lower body weight on day 7 (8.42±0.83 g vs 5.78±1.08 g, P =0.002) , on day 14 (15.56±1.23 g vs 8.32±1.33 g, P=0.000) , and on day 21 (25.26±1.83 g vs 12.72±1.28 g, P =.000), but not on day 3 (3.88±0.46 g vs 3.52±0.37 g, P =0.200). (Fig. 1)

Structural changes were evident in the hyperoxia and control mice lungs on day 7, day 14, and day 21. At 3 days the hyperoxia mice had no obvious qualitative differences in the lung regions characterized by either large distal air spaces or absence of interstitial thickening. However, at 7 days the hyperoxia mice had heterogeneous lung structure changes, including patchy areas of parenchymal thickening, small air spaces interspersed in enlarged air spaces. At 21 days the hypoxia mice had a lung injury pattern that has morphologic similarities to human BPD (Fig. 2).

Compared to air control group, hyperoxia reduced radial alveolar count significantly on day 7 (6.31 ±0.97 vs 5.12±1.72, P =0.000), day 14(6.96±0.97 vs 5.92±1.48, P =0.000) and day 21(10.08± 1.27 vs 7.84±1.13, P =0.000), but not on day 3 (4.17±0.83 vs 4.05±1.15, P =0. 464). (Fig. 3) The alveolar enlargement and decrease in surface area are associated with decreased oxygen transfer and arterial oxygen saturation, resulting in the characteristic BPD-related reduction in lung function.

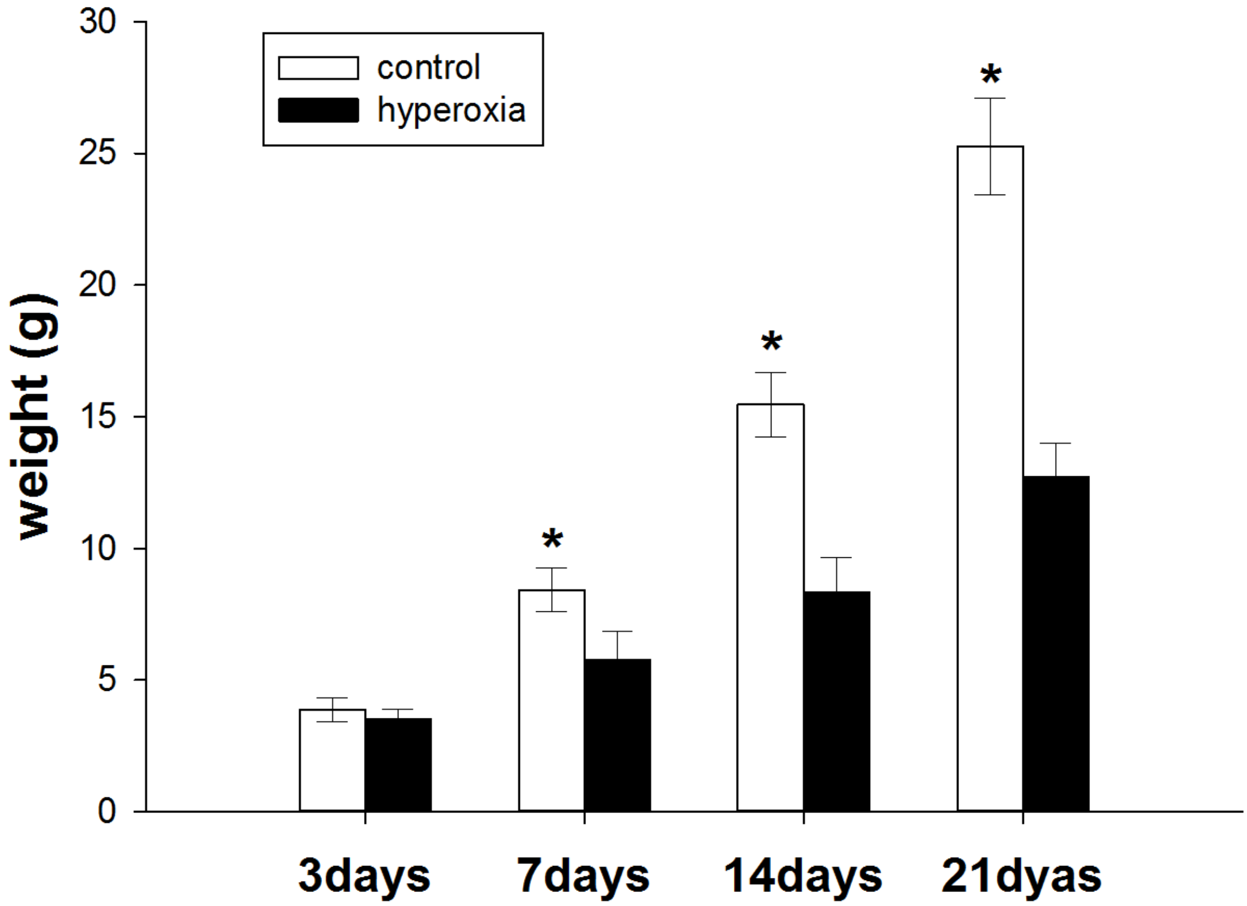

Fig. 1 Effects of 60% Oxygen Exposure on Body weight of mice (n=5 per group; *P<0.05)

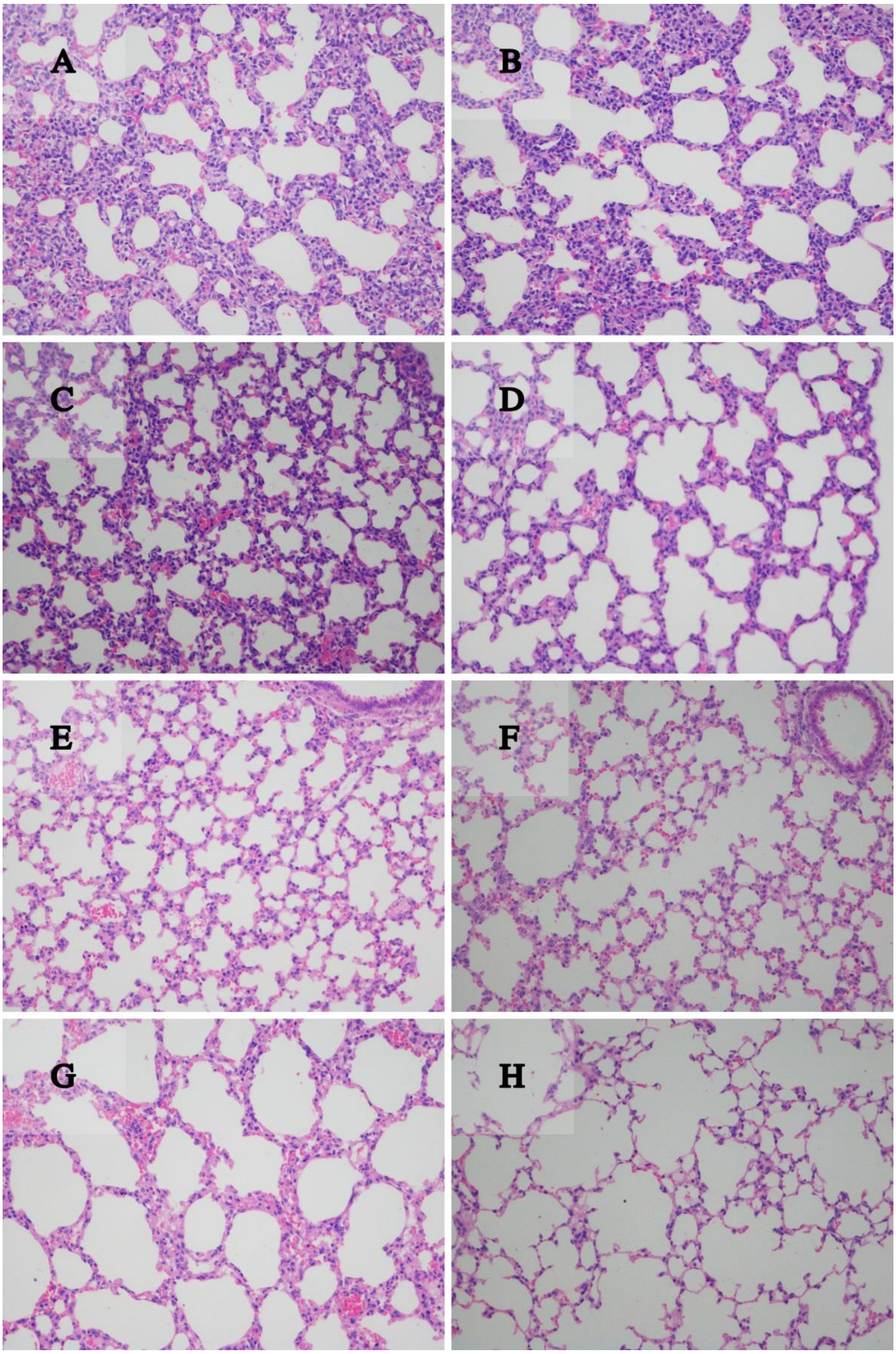

Fig. 2 Lung structural changes of mice induced by 60% Oxygen Exposure

A: air control group on day 3 (100×); B: hyperoxia + PBS group on day 3 (100×); C: air control group on day 7 (100×); D: hyperoxia + PBS group on day 7 (100×); E: air control group on day 14 (100×); F: hyperoxia + PBS group on day 14 (100×); G: air control group on day 21 (100×); H: hyperoxia + PBS group on day 21 (100×).

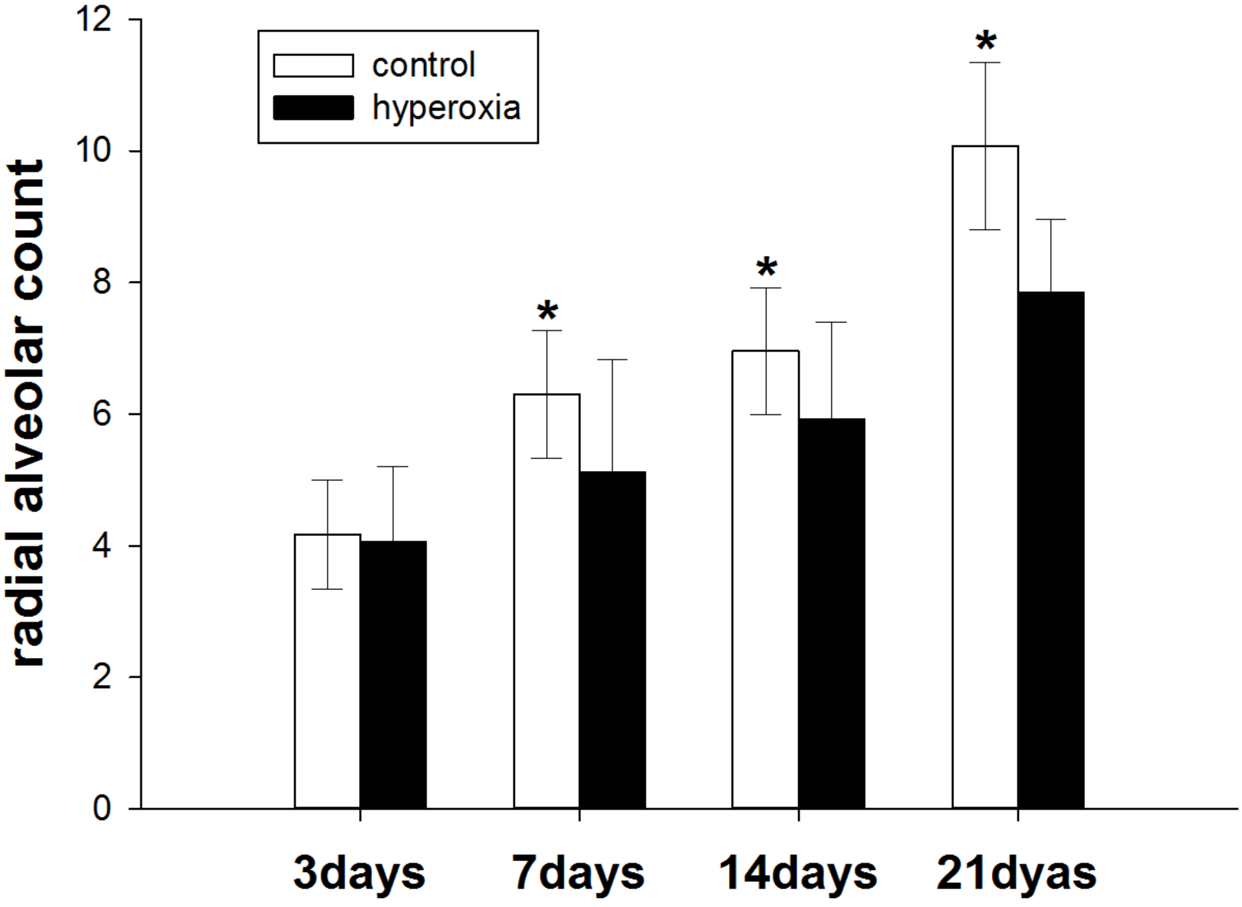

Fig. 3 Effects of 60% Oxygen Exposure on RAC of mice (n=5 per group; *P<0.05)

Part 2. Identification of L-MSCs

Methods

In vitro culture and identification of neonatal mouse L-MSCs

The lung tissues were collected from neonate KM mice under an aseptic condition, then digested with collagenase and cultured in vitro. Flow cytometry was employed for the detection of surface markers (CD29, CD105, CD73, CD90, Scal-1, CD34 and CD44). Osteogenic and adipogenic inductions were performed with corresponding kits to investigate the differential potentials of these cells. These cells were passaged, frozen, and thawed after collection, and then cell morphology was observed.

1 Separation and culture of L-MSCs

The lung tissues were collected from neonatal KM mice at 1-2 days after birth, the tracheal and bronchial tissues were removed, and the lung tissues were washed with PBS to remove blood cells and then cut into pieces (1 mm^3). 0.2 g of lung tissues was collected and mixed with 5 ml of 0.2% collagenase II in PBS at 37℃, followed by digestion for 30 min. The resultant tissues were pipetted repeatedly, followed by additional digestion for 15 min. The resultant solution was filtered through a 200-mesh filter and then a 100-mesh filter (if necessary, the solution can be diluted). The filtrate was centrifuged at 1500 rpm for 10 min, and cells were harvested. At room temperature, cells were re-suspended at a ration of 1:3-5 and then incubated at room temperature for 5 min. Incubation was stopped by addition of PBS of equal volume. The cell suspension was filtered through a 100-mesh filter, followed by cell counting. Cells were re-suspended in complete DMEM/F12 medium (10% FBS, 100 U/ml penicillin, 100 μg/ml streptomycin, 2.0 mM L-glutamine, 10 mM HEPES, 37℃), and the cell density was adjusted to 1×10^6 cells/ml. Cells were seeded into flasks at 5×10^4 cells/ml, followed by incubated at 37 ℃ in a humidified environment. On the second day (>16 h), a half medium was refreshed to remove suspended cells, and then the medium was refreshed once every 2-3 days. When the cell confluence reached 70-80%, cells were digested with 0.25% trypsin in 0.02% EDTA and collected as primary L-MSCs.

When the cell confluence reached 70-80%, cells were digested with 0.25% trypsin in 0.02% EDTA for passaging. In this study, L-MSCs of passage 2-6 were used. The cell number and viability were

determined by trypan blue staining; a cell viability percentage of at least 95% was required for all of the tests.

2 Identification of surface markers by flow cytometry

L-MSCs of passage 2-4 were digested and collected, followed by washing with PBS. After centrifugation at 1000 rpm for 5 min, the supernatant was removed. Cells were re-suspended in 100 μl of PBS, followed by addition of PE-CD29, PE-CD105, FITC-CD73, FITC-CD90, PE-Scal-1, FITC-CD34, FITC-CD44 and FITC/PE mouse IgG1 isotype (sigma, USA). After incubation at 4℃ for 30 min in dark, cells were washed in PBS by centrifugation at 1000 rpm for 5 min, and the supernatant was removed. Cells were re-suspended in 400 μl of loading buffer for flow cytometry (FC 500; BecmanCouiter, USA).

3 Osteogenic, adipogenic and chondrogenic inductions of L-MSCs

Adipogenic/osteogenic induction was performed with corresponding kits (Cyagen, USA) according to manufacturer's instruction.

Results

1. Morphology of L-MSCs after separation

At 24 h after seeding, cells were adherent to the bottom, and these cells were mainly round. After 2 days, some cells displayed protrusions, and there were spindle-shaped cells and small round cells; the spindle-shaped cells were irising. After 10-15 days, cell number increased significantly, and cells showed whirlpool-like distribution. In addition, there were a few round epithelial cells around the spindle-shaped cells which were removed after refreshing medium. The passaging several times had no influence on the cell morphology, the cell morphology also remained after being thawed, and cells showed good growth (Figure 1).

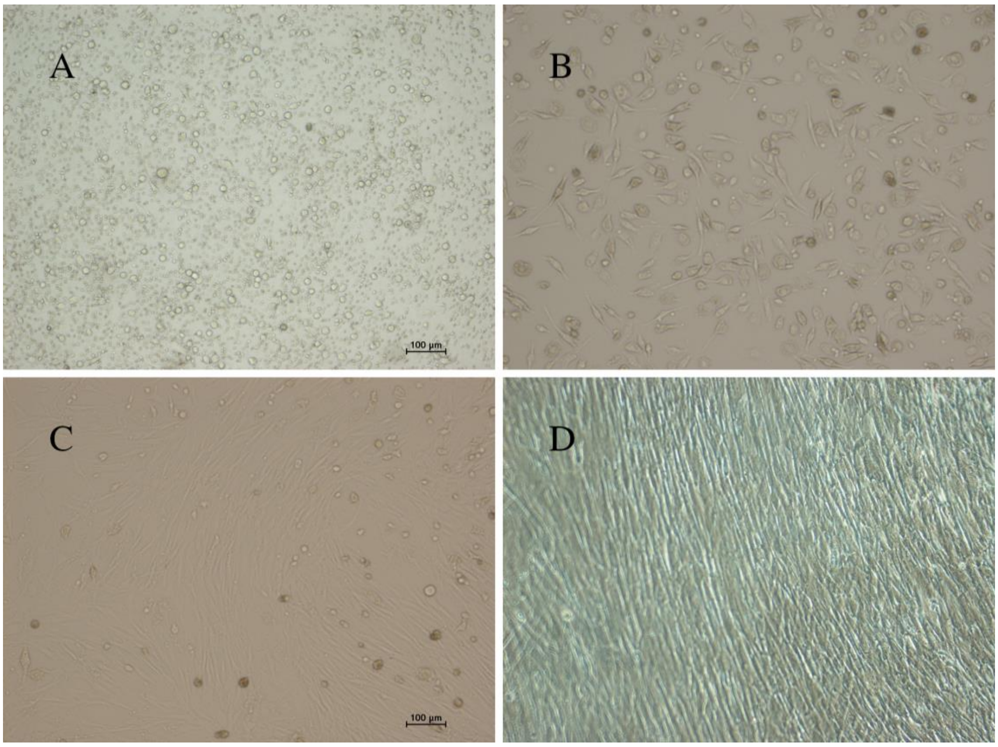

Figure 1.Morphology of L-MSCs after seeding.

A: 24 h; B: 3 days; C: P0; D: P3；Scale bar=100 μm and 4 pictures have the same magnification

2. Surface markers of L-MSCs

Flow cytometry analysis was performed for the detection of surface markers. Results showed L-MSCs were positive for CD105, CD73, CD90, Scal-1 and CD29, but negative for CD34 and CD44 (CD29 expression: not shown) (Figure 2). This was consistent with the phenotype of MSCs.

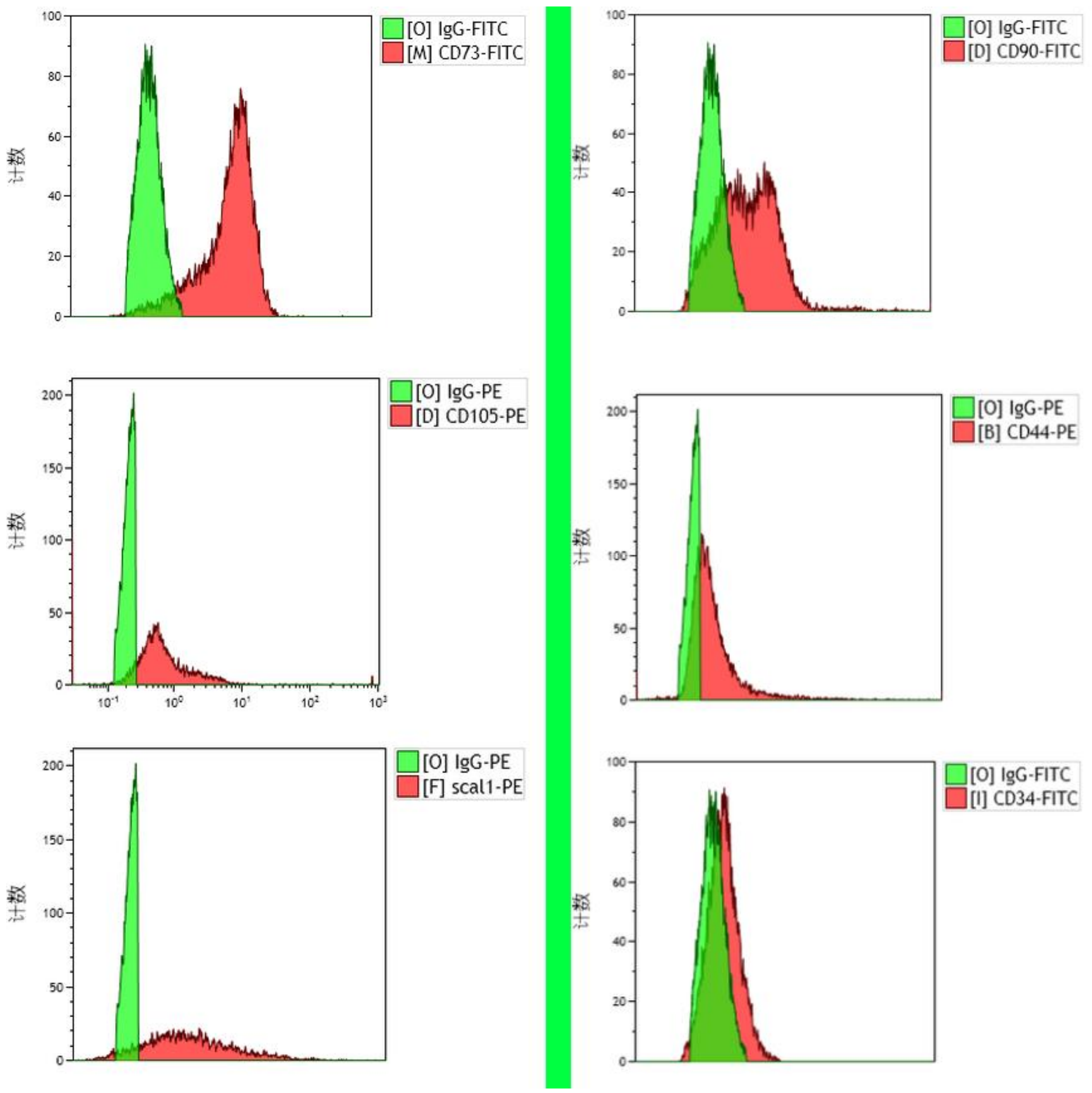

Figure 2. Flow cytometry analysis of surface markers of L-MSCs

Flow cytometry analysis shows L-MSCs were positive for CD73, CD90, CD105 and Scal-1, but negative for CD34 and CD44.

3. Osteogenic induction of L-MSCs

When the cell confluence reached 70%, cells received osteogenic induction. At 4 days after induction, cell volume increased, cells became polygonal, and cytoplasmic granules increased; at 8 days after induction, cells became paving-stone-like, the cytoplasm was full of granules, and there was calcium deposition between cells; at 11 days after induction, calcium nodules merged to form multiple mineralization centers which were red after Alizarin red staining. In the control group, this phenomenon was not observed. These indicate that osteogenic induction is successful (Figure 3).

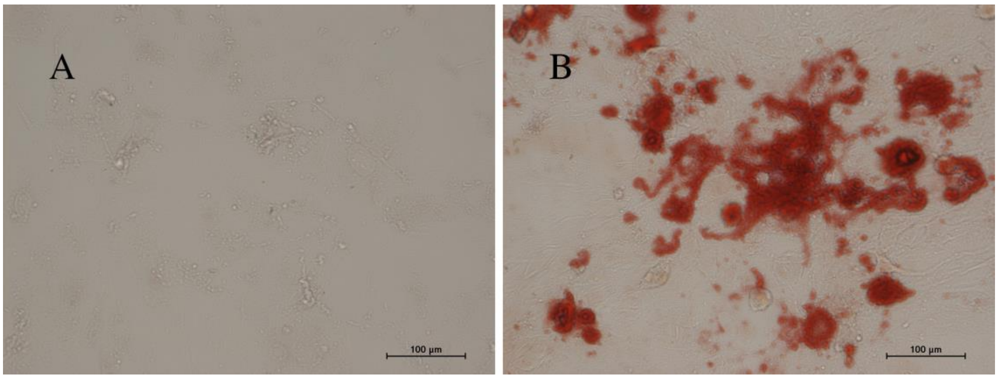

Figure 3.Osteogenic induction of L-MSCs.

A: negative control (200×); B: L-MSCs at 11th day after induction (200×). Scale bar=100 μm, and Alizarin red staining shows the calcium nodules formation in induced L-MSCs (B).

4. Adipogenic induction of L-MSCs

Cells underwent adipogenic induction after cell confluence reached 70-80%. At 4 days, lipid droplets were observed and merged subsequently; cells became polygonal; a large amount of lipid deposition was observed in cells after oil red O staining. These indicate that adipogenic induction is successful (Figure 1.4).

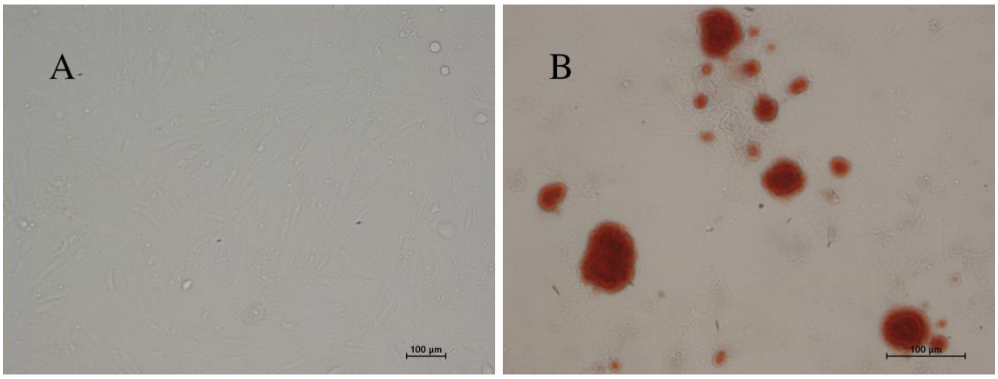

Figure 4.Adipogenic induction of L-MSCs.

A: negative control (100×); B: L-MSCs at 10th day after induction (200×). Scale bar=100 μm, and oil red O staining shows a large amount of lipid deposition in induced L-MSCs.
